# Health Foods in Philadelphia

**Published:** 1879-08

**Authors:** 


### *HEALTH FOODS IN PHILALELPHIA.*

An agency for the Health Food Company's preparations has lately been established in Philadelphia, at No. 632 Arch street, by Mr. George I. Wilson, formerly of Jersey City. Mr. Wilson brings to this excellent work admirable business ability, a knowledge of physiology and practical hygiene, and a firm conviction, founded on personal observation and experience, that these admirable foods are the one thing needful in all remedial agencies. We commend the Company and its agent to our Philadelphia readers.

Our friends in Germantown will be supplied with the Health Foods by Messrs. Jno. T. Roberts & Bro., as heretofore.

In Toronto the Health Food Company have as agents two enthusiastic and accomplished ladies, Drs. Trout and Tefft, to whom our Canadian readers should apply.

HEALTH FOOD CO.—We have been too busy to allude to the noble products of this Company for many months, and we undertake to make up for past remissness by an exhaustive treatment of the whole subject in this issue. It is hardly possible to say too much for the good work which they are doing. It is easy to treat the sick who are nourished and supported by these strengthening foods.

